# Contribution of the Technical Efficiency of Public Health Programs to National Trends and Regional Disparities in Unintentional Childhood Injury in Japan

**DOI:** 10.3389/fpubh.2022.913875

**Published:** 2022-07-12

**Authors:** Ayumi Hashimoto, Hiroyuki Kawaguchi, Hideki Hashimoto

**Affiliations:** ^1^Graduate School of Medicine, University of Tokyo, Tokyo, Japan; ^2^Economics Faculty, Seijo University, Tokyo, Japan; ^3^Department of Health and Social Behavior, School of Public Health, University of Tokyo, Tokyo, Japan

**Keywords:** efficiency, public health, injuries, child, Japan

## Abstract

To achieve the Sustainable Development Goals, strengthening investments in health service inputs has been widely emphasized, but less attention has been paid to tackling variation in the technical efficiency of services. In this study, we estimated the technical efficiency of local public health programs for the prevention of unintentional childhood injury and explored its contribution to national trend changes and regional health disparities in Japan. Efficiency scores were estimated based on the Cobb–Douglas and translog production functions using a true fixed effects model in a stochastic frontier analysis to account for unobserved time-invariant heterogeneity across prefectures. Using public data sources, we compiled panel data from 2001 to 2017 for all 47 prefectures in Japan. We treated disability-adjusted life years (DALYs) as the output, coverage rates of public health programs as inputs, and caregivers' capacity and environmental factors as constraints. To investigate the contribution of efficiency to trend changes and disparities in output, we calculated the predicted DALYs with several measures of inefficiency scores (2001 average, yearly average, and prefecture-year-specific estimates). In the translog model, mean efficiency increased from 0.62 in 2001 to 0.85 in 2017. The efficiency gaps among prefectures narrowed until 2007 and then remained constant until 2017. Holding inefficiency score constant, inputs and constraints contributed to improvements in average DALYs and widened regional gaps. Improved efficiency over the years further contributed to improvements in average DALYs. Efficiency improvement in low-output regions and stagnated improvement in high-output regions offset the trend of widening regional health disparities. Similar results were obtained with the Cobb–Douglas model. Our results demonstrated that assessing the inputs, constraints, output, and technical efficiency of public health programs could provide policy leverage relevant to region-specific conditions and performance to achieve health promotion and equity.

## Introduction

The child health burden has dramatically reduced in the last three decades. Total disability-adjusted life years (DALYs) for those aged under 20 years decreased by 46% from 1990 to 2017, dropping from 1.31 billion to 709 million. This decline was led by drastic improvements in communicable, maternal, neonatal, and nutritional diseases in low- and middle- income countries (LMICs) (1, 2). Further improvement can be expected as universal health coverage increases and economic growth proceeds. However, in LMICs, injuries have grown in relative importance for total DALYs from 1990 to 2017, climbing from 5.7 to 6.5% in low-income countries and from 12.6 to 14.0% in middle-income countries, in contrast to a drop from 19.9 to 13.5% in high-income countries. Nonetheless, unintentional injury has been a leading cause of health burden among children in high-income countries (3–5), suggesting that socioeconomic development does not necessary solve these problems. Public health interventions targeting parenting quality are essential to prevent unintentional injuries, especially for preschool children, an effort that is likely to be resource consuming. To achieve the Sustainable Development Goals (SDGs), in addition to strengthening investments in health service inputs, the question of how to prepare high-quality public health programs in an efficient manner will be key (6).

Parental health education is known to be an effective vehicle for preventing unintentional childhood injury; health education enhances parental supervision and environmental preparedness at home in terms of using safety equipment and removing home hazards (7–11). Mental health support for mothers with depression or high levels of stress also has the potential to reduce unintentional childhood injuries resulting from poor parenting quality (9, 12, 13). Moreover, community-based interventions for safety such as public campaigns and infrastructure management have been caried out (14, 15).

There are several potential providers of these interventions, including health care institutions, schools, and private organizations (9–11, 14–16). Unlike most Organization for Economic Co-operation and Development (OECD) countries, where primary care practitioners are responsible for delivering public health services, Japan relies on a public health division in regional government offices to play a major role in administering its legally established free public health programs (17–19). Specifically, the prefecture-level public health divisions administer the public health programs, and program operation is conducted by municipal level centers to better meet the needs of local communities. Under the nationwide standardized protocol that designates which types of programs are to be provided for preschool children and their caregivers, Japan's decentralized system allows local discretion in priority setting and budgeting (17) so that local authorities can choose the size and frequency of service provision. Program operations regarding the mode of target population segmentation, health needs assessment, and selection of health education content are locally decided (20–22).

Although childhood injury mortality has declined nationally, there are considerable geographic disparities across Japan (23, 24). Given the relatively high educational attainment (25) and affordable universal health coverage since 1961 (26), the change in Japan's national trends and remaining geographic disparities may not be explained solely by local demographic characteristics and environmental uniqueness (27). Instead, investment in public health programs and changes in their effectiveness may provide an alternative explanation, with important policy implications. However, previous studies have failed to distinguish between the contributions of input, constraint conditions, and technical efficiency in the assessment of the effectiveness of public health programs (14, 15).

In this study, we estimated the technical efficiency of public health programs aiming to prevent unintentional childhood injury in Japan, and we distinguished the contribution of technical efficiency to national trend changes and regional health disparities from those of input and constraints. Overall cost efficiency is a product of technical efficiency and allocative efficiency; here, we focused specifically on technical efficiency because it reflects the current state of technology with regard to potentially producing the maximum output attainable at each input level (28, 29). Because the human and financial resources available for public services are often limited, we considered separate assessments of inputs, constraints, and technical efficiency promising in terms of providing policy leverage for improving and equalizing health outcomes across regions.

In the following section, we briefly review previous studies that have estimated technical efficiency in health, before presenting a theoretical model of the production function of unintentional childhood injury. We relied on stochastic frontier analysis (SFA) and a true fixed effects model (TFEM) to obtain efficiency estimations, accounting for time-invariant local heterogeneity. Our estimation revealed the existence of disparities. We also showed different trajectories of improvement in technical efficiency over time, which contributed to narrowing geographic health disparities. The final section discusses several policy implications for the SDGs, including closing the health gap.

## Materials and Methods

### Short Review of Technical Efficiency in Health

In health economics, there are two categories of production function—namely, the production function of *health care* and the production function of *health* (30, 31). A number of studies have concentrated on the production function of health care in hospitals and nursing homes (32–37), whereas the production function of health has been used for the evaluation of national health systems' performance (38–42).

In the production function of health care, the output is usually defined as the number of treated cases, and the inputs are physical capital investment (e.g., the number of beds and other medical equipment) and the health care labor force (e.g., the number of physicians and nurses). In the production function of health, the output is health status, and the inputs are health care services, along with demographic characteristics, personal health habits, and environmental factors.

Using the production function of health, in 2000, the World Health Organization published a league table ranking the efficiency of the health systems of 191 countries (38, 39), which was criticized for ignoring heterogeneity among OECD countries and non-OECD countries (40, 41). A recent meta-analysis of cross-country comparisons of OECD countries revealed inconsistent country rankings on efficiency and argued that the lack of a theoretical foundation for the production function of health systems may cause this inconsistency (42).

Another potential explanation for these inconsistent results may be found in the lack of differentiation of curative medical care and preventive public health services. Compared with curative medical care, preventive services may have a larger influence on certain health consequences when the effectiveness of *post-hoc* medical intervention is limited. Unintentional injury is such a case. Medical care after children sustain serious injuries has limited effectiveness in terms of reducing mortality and morbidity because most trauma deaths (i.e., 57–85%) occur before hospitalization (43–45), and the in-hospital mortality of pediatric trauma patients has remained around 4–7% in developed countries (43, 46, 47). Moreover, seriously injured children often have lifelong disabilities (48, 49). Therefore, the prevention of the initial injury-causing event is a major way to effectively reduce mortality and morbidity from injury among children.

Although public health services play a major role in promoting population health by preventing diseases and injuries, studies on these services' technical efficiency are scarce (32, 33). Studies on local public health departments in China and the United States exist, but they have only measured the production function of health care (50, 51). To our knowledge, no studies have estimated the technical efficiency of public health programs using the production function of health.

### Theoretical Model of the Production Function of Unintentional Childhood Injury

Preventive services achieve health improvement through increased health-related knowledge and the subsequent modification of individual attitudes and targeted behaviors in the targeted population. These achievements are known to be affected by the quality of the education program, the receivers' health literacy, and the supportive environment (52–59).

Considering the above points, we defined the production function of unintentional childhood injury as a structural equation:


(1) $$\begin{array}{r} Y=f\left(X,Z\right) \end{array}$$

The output of the production function (*Y*) denotes population health outcomes related to unintentional childhood injury. *Y* is assumed to be a function of *X*, which represents inputs, in addition to the constraining factors (Z). More specifically, *X* denotes the volume of public health programs on preventing unintentional childhood injury. Public health programs potentially modify caregivers' knowledge, attitudes, and behaviors (7–11). Z denotes the caregivers' capacity and environmental factors. For the caregivers' capacity, we assume that caregivers' educational level and occupational status reflect their health literacy (55–57). In terms of environmental factors, traffic and the natural environment are related to the occurrence of unintentional childhood injury (57, 58). Access to emergency medical care providing specialized treatment for trauma patients is assumed to improve survival rates and functional outcomes (47, 59).

### Empirical Model to Estimate Technical Efficiency

We used SFA rather than nonparametric data envelopment analysis because SFA can account for random error. SFA is more robust to outlying observations and changes in the input and output variables, compared with data envelopment analysis (28, 60). Nevertheless, SFA-estimated efficiency is still confounded by heterogeneity across samples. Greene has proposed the TFEM (40, 61), which can distinguish unobserved time-invariant heterogeneity across samples from estimated efficiency. Moreover, the TFEM assumes that efficiency is time-variant without assuming any functional form of time trend or the same time trend across samples. We believed that this assumption better reflected the reality of local technical efficiency.

For functional form, we adopted the conventional Cobb–Douglas form, as shown in equation (2), and the more flexible translog form, as shown in Equation (3):


(2) $$\begin{array}{rc} lnY_{it} & =\alpha_{i}+\beta_{1}lnX_{it}+\beta_{2}\ln\;Z_{it}+v_{it}-u_{it}, \end{array}$$

(3) $$\begin{array}{l} lnY_{it}=\alpha_{i}+\beta_{1}lnX_{it}+\frac{1}{2}\beta_{2}{\left(lnX_{it}\right)}^{2}\\ \;+\beta_{3}\left(lnX_{it}\right)\left(ln{X^{\prime }}_{it}\right)+\beta_{4}lnZ_{it}\\ \;+v_{it}-u_{it},\\ \;v_{it}\sim N\left[0,\sigma^{2}{}_{v}\right],\;u_{it}=\left|U_{it}\right|,\\ \;U_{it}\sim N\left[0,\sigma^{2}{}_{u}\;\right]. \end{array}$$

Here, *Y, X*, and *Z* are the output (the health outcome of unintentional childhood injury), input (the volume of public health programs), and constraining factors (caregivers' capacity and environmental factors), respectively. Prefecture is denoted by *i*, year is denoted by t, and the random error term is denoted by v_it_. *u*_*it*_ is the inefficiency term, which is assumed to be half-normally distributed. α_i_ is the time-invariant prefecture-specific fixed effect, which may represent unmeasurable environmental factors such as the safety culture in communities. *Y, X*, and *Z* were natural log-transformed. Some variables were linearly transformed before being natural log-transformed for the model conversion. We assumed no time lag between input and output because we supposed that the major vehicle for reducing injury risk was parental behavioral change, which should happen in a short time (10, 22). The parameters were estimated by maximum likelihood. The efficiency score was estimated using the estimator proposed by Jondrow et al. (62). The “sfpanel” command with the “model (tfe)” option in Stata 16.0 (StataCorp, College Station, USA) was used for the analyses (63).

As a robustness check, we estimated technical efficiency using models assuming different distributions of the inefficiency terms (i.e., exponential and truncated-normal distributions) (40, 61, 63) and models assuming interactions between the input (X) and constraining factors (Z), as shown in Equation (4):


(4) $$\begin{array}{l} lnY_{it}=\alpha_{i}+\beta_{1}lnX_{it}+\frac{1}{2}\beta_{2}{\left(lnX_{it}\right)}^{2}+\beta_{3}\left(lnX_{it}\right)\left(ln{X^{\prime }}_{it}\right)\\ \;+\beta_{4}lnZ_{it}+\beta_{5}\left(lnX_{it}\right)\left(lnZ_{it}\right)+v_{it}-u_{it} \end{array}$$

We also estimated technical efficiency excluding the areas damaged by the Great East Japan Earthquake (i.e., Miyagi, Fukushima, and Iwate).

### Sample and Variables for the Efficiency Estimation Model

Panel data were compiled from 2001 to 2017 for all 47 prefectures in Japan (*n* = 799) because prefectures are the legislative units for public health policy in Japan. For the output (*Y*), prefecture-level numbers of DALYs by age, cause, and year were obtained from the Global Burden of Disease Study 2017 (2). The number of DALYs is a composite measure of disease burden that reflects both premature mortality and the prevalence and severity of ill health; it is the sum of the years of life lost and the years lived with disability (27, 64). In this study, we used linearly transformed DALYs for unintentional injury per 100,000 population aged under 5 years as the output; higher output values indicate better production of health. More specifically, we subtracted the number of DALYs from the median DALYs and linearly transformed the result so that the minimum value would be one. Population numbers were obtained from the population census and official population estimates (25, 65). Unintentional injury was categorized as pulmonary aspiration or foreign body in the airway; falling; drowning; fire, heat, or hot substances; transport injury; or other unintentional injury. Injuries caused by exposure to forces of nature were excluded to remove the influence of the Great East Japan Earthquake that hit the northeastern regions of Japan in 2011.

In terms of inputs (*X*), we compiled the prefecture-level coverage rates of all public health programs related to childhood injuries for which provision is not mandatory but is strongly recommended by national laws—namely, health checkups for children aged under 1 year, home visits for children aged under 1 year (i.e., home visits for newborns, premature babies, and children aged under 1 year), health guidance at the individual or group level, and health education at the group or community level (13, 14, 17, 20–22, 66, 67). We excluded some legally mandated services (i.e., health checkups for children aged 1.5 years and 3 years) from the list of inputs because nearly all children in all prefectures undergo these health checkups, and strictly standardized programs are less likely to contribute to regional variation in technical efficiency.

Trained public health nurses are the main providers of these programs, which are intended to enhance parental supervision and environmental preparedness. The main purposes of the health checkups are monitoring child growth and providing caregivers with childcare knowledge. Home visits are conducted to support caregivers to reduce behavioral risks based on an assessment of the home environment. Health guidance at the individual or group level is defined as guidance on specific topics such as child health, nutrition, and exercise. Health education at the group or community level is defined as health promotion activities to improve maternal and child health, mainly targeting women of reproductive age.

The coverage rate of each program was calculated based on the program characteristics by dividing the number of program participants by the target population size, as described in Table 1. The data on public health programs were obtained from government reports that all local governments are required to submit each fiscal year (66). We replaced only one missing observation for health checkup with the value from the previous year. Because the report for fiscal year 2010 did not include data from municipalities that were affected by the Great East Japan Earthquake in Iwate, Miyagi, and Fukushima prefectures, coverage rates for these prefectures in 2010 were calculated without the affected municipalities.

**Table 1:** Descriptive statistics of output, input, and constraining factors from 2001 to 2017 (*n* = 799).

**Variable**	**Measurement**	**Mean**	**Standard deviation**	**Minimum**	**Maximum**
**Output variable**					
Unintentional injury^a^	DALYs^b^ per 100,000 population aged under 5 years	877.5	155.3	496.3	1307.9
Pulmonary aspiration or foreign body in the airway		279.7	57.7	153.3	439.9
Falling		118.1	8.9	92.8	162.2
Drowning		136.8	42	48.9	287.8
Fire, heat, or hot substances		53.8	13.7	23.8	100.1
Transport injury		157.9	47.4	57.8	337.1
Other unintentional injury		131.1	11	107	170.4
**Input variables**					
**Coverage rates of public health programs** ^**c**^					
Health checkups for children aged under 1 year	Proportion of children aged under 1 year who received health checkups^d^	0.57	0.13	0.33	1
Home visits for children aged under 1 year	Proportion of children aged under 1 year who received home visits	0.68	0.27	0.18	1.42
Health guidance at the individual or group level	Proportion of children aged 0–4 years whose caregivers received health guidance	1.35	0.31	0.52	2.59
Health education at the group or community level	Proportion of women aged 15–49 years receiving health education	0.11	0.04	0.01	0.32
**Constraining factors**					
Proportion of the population with tertiary education	Proportion of the population aged 20–49 years with complete college, university, or graduate school	0.37	0.06	0.24	0.49
Unemployment rate	Number of people actively looking for a job as a proportion of the total labor force	0.04	0.01	0.01	0.08
Density of emergency medical facilities	Number of tertiary emergency medical facilities per 100,000 population	0.2	0.09	0.05	0.58
Population density	Number of people per 1 km^2^	649.6	1,142.6	68.6	6,168.7
Great East Japan Earthquake dummy	1: Iwate, Miyagi, or Fukushima prefectures in 2011, 0: other	–	–	–	–

^a^*Injuries by exposure to forces of nature were excluded from the analysis*.

^b^*DALYs: disability-adjusted life years*.

^c^*Coverage rates were calculated by dividing the number of participants by the target population size. The coverage rate could exceed one because the cumulative total number (i.e., health guidance and health education) or the sum of the actual number (i.e., home visits) of participants was used as the numerator*.

^d^*The target population was defined as four times the number of children aged under 1 year because the maximum number of health checkups was four. For the numerator, the sum of the actual number of participants in four health checkups was used from 2005 to 2017, whereas the total number of participants in health checkups was used from 2001 to 2004 because of data availability*.

In terms of caregivers' capacity (*Z*), the regional education level and unemployment rate were used as indicators of health literacy. The regional education level of the child-rearing generation was measured as the proportion of people aged 20–49 years in the total population who had completed tertiary education, which was derived from the 2000 and 2010 Population Censuses and allocated to 2001–2009 and 2010–2017, respectively (25). The region-level (i.e., set of prefectures) annual unemployment rate was obtained from the Labour Force Survey (68) and allocated to each corresponding prefecture.

Several environmental factors (*Z*) were assessed. To gauge access to emergency medical care including specialized treatment for trauma patients, the number of tertiary emergency medical facilities per 100,000 population was obtained from the Survey of Medical Institutions (69). Data from 1999, 2002, 2005, 2008, 2011, 2014, and 2017 were allocated to 2001, 2002–2004, 2005–2007, 2008–2010, 2011–2013, 2014–2016, and 2017, respectively. Data from 2011 were missing for some areas damaged by the Great East Japan Earthquake (i.e., Miyagi and Fukushima); thus, we allocated the values in the 2014 data to 2011–2016 for these areas. Population density, which was assumed to represent the level of traffic and the natural environment, was obtained from the Population Census (25). Data from 2000, 2005, 2010, and 2015 were allocated to 2001–2004, 2005–2009, 2010–2014, and 2015–2017, respectively. Because the Great East Japan Earthquake in 2011 severely damaged the living environment and the overall public health system in Iwate, Miyagi, and Fukushima prefectures (70), a dummy variable for these three affected prefectures in 2011 was also included.

### Contributions of Inputs, Constraints, and Technical Efficiency to National Trends and Regional Disparities in Unintentional Childhood Injury

To investigate the contributions of inputs, constraints, and technical efficiency to average changes and disparities in DALYs, we compared the time trend of predicted DALYs using three types of inefficiency scores. First, to examine only the influences of inputs and constraining factors on DALYs, we predicted the counter-factual DALYs, holding the inefficiency score constant at its mean value for 2001 (i.e., Σ*u*_*it*_/47; *t* = 2001) and leaving the fixed effects of prefecture, inputs, and constraints as they were in Equations (2) and (3). Second, to investigate the influence of technical efficiency trends, we predicted the DALYs using the annual mean score of inefficiency (i.e., Σ*u*_*it*_/47; *t* = 2001–2017). Finally, we prepared the predicted DALYs using the actual prefecture-year-specific inefficiency estimates (i.e., *u*_*it*_).

By comparing these three sets of predicted DALYs, we were able to distinguish national trends and geographic disparities attributable to inputs, constraining factors, and the fixed effects of prefecture from those attributable to technical efficiency. We used the coefficient of variance (CV; the standard deviation of the value divided by the mean) to summarize the magnitude of regional disparities in the examined variables.

Because our study involved secondary analysis of anonymous data, the requirement for ethical approval and informed consent was waived under governmental-use approval and under the approval of the Institute for Health Metrics and Evaluation of the University of Washington for use of the Global Burden of Disease Study 2017 by non-commercial users.

## Results

### Descriptive Statistics of Variables Used to Estimate Technical Efficiency

Table 1 shows the descriptive statistics of the output, input, and constraining factors. The mean DALYs for unintentional injury per 100,000 population aged under 5 years decreased from 1,097.4 in 2001 to 698.1 in 2017. Similar time trends in DALYs were found across the 47 prefectures. The disparities in DALYs among prefectures did not narrow during the study period; the standard deviation of DALYs was 109.2 in 2001 and 90.5 in 2017, and the CV of DALYs was 0.10 in 2001 and 0.13 in 2017. The mean years of life lost and the mean years lived with disability because of unintentional injury were 539.6 and 158.6, respectively, in 2017. On average, all inputs and constraining factors increased from 2001 to 2017, except for unemployment rate, which decreased. For most inputs and constraining factors, the prefectural disparities measured by the standard deviation remained constant during the study period.

### Estimation of Technical Efficiency

Following the results of the *F*-test in the pooled ordinary least squares model (*P* < 0.0001) and the Hausman test to select between the fixed effects regression model and the random-effects generalized least squares regression model (*P* < 0.0001), TFEM was selected to estimate technical efficiency in the Cobb–Douglas and translog models. The correlations between the fixed effect terms and estimated technical efficiency by year ranged from −0.15 to 0.29 in the Cobb–Douglas model and from −0.15 to 0.42 in the translog model. These correlations were not statistically significant except in 2001 in the Cobb–Douglas model and in 2001, 2010, and 2012 in the translog model (*P* < 0.05), which suggests no or low correlations between the two estimates.

The TFEM estimations are presented in Table 2. In the Cobb–Douglas model, all variables were statistically significant. For the inputs, the coefficients of the coverage rates of home visits, health guidance, and health education were positive, as expected, which suggests that these programs decreased injury burden. Only the coefficient of the coverage rate of health checkups was negative. Turning to the constraining factors, a positive coefficient of the proportion of the population with tertiary education and negative coefficients of unemployment rate and the Great East Japan Earthquake dummy were found as expected. Contrary to our expectations, the coefficients of density of emergency medical facilities and population density were negative, although the coefficient of emergency medical facilities was small.

**Table 2:** Estimated production function using a true fixed effects model (*N* = 47 for 17 years)^a^.

	**Cobb–Douglas production function**				**Translog production function** ^**b**^			
	**Coefficient**	**95% confidence intervals**		***P* > |*Z*|**	**Coefficient**	**95% confidence intervals**		***P* > |*Z*|**
		**Lower limit**	**Upper limit**			**Lower limit**	**Upper limit**	
**Coverage rates of public health programs**								
ln Health checkups for children aged under 1 year	−0.60	−0.69	−0.5	<0.001	−0.35	−0.4	−0.3	<0.001
ln Home visits for children aged under 1 year	0.5	0.49	0.52	<0.001	0.5	0.46	0.53	<0.001
ln Health guidance at the individual or group level	0.27	0.26	0.29	<0.001	0.27	0.12	0.42	<0.001
ln Health education at the group or community level	0.07	0.04	0.1	<0.001	0.12	0.08	0.16	<0.001
ln Proportion of the population with tertiary education	0.21	0.11	0.31	<0.001	0.7	0.58	0.81	<0.001
ln Unemployment rate	−0.25	−0.29	−0.22	<0.001	−0.21	−0.25	−0.18	<0.001
ln Density of emergency medical facilities	−0.01	−0.01	0	0.04	0.04	−0.01	0.1	0.13
ln Population density	−1.59	−2.37	−0.82	<0.001	−0.76	−0.82	−0.69	<0.001
Great East Japan Earthquake dummy	−0.04	−0.08	0	0.03	−0.24	−0.32	−0.16	<0.001
ln Health checkups for children aged under 1 year (squared)					−2.18	−2.86	−1.49	<0.001
ln Home visits for children aged under 1 year (squared)					−0.15	−0.22	−0.08	<0.001
ln Health guidance at the individual or group level (squared)					−0.13	−0.37	0.12	0.31
ln Health education at the group or community level (squared)					0.17	0.02	0.32	0.02
ln Health checkups for children aged under 1 year * ln Home visits for children aged under 1 year					0.8	0.57	1.02	<0.001
ln Health checkups for children aged under 1 year * ln Health guidance at the individual or group level					−0.54	−1.1	0.02	0.06
ln Health checkups for children aged under 1 year * ln Health education at the group or community level					0.26	0.03	0.49	0.03
ln Home visits for children aged under 1 year * ln Health guidance at the individual or group level					0.56	0.45	0.68	<0.001
ln Home visits for children aged under 1 year * ln Health education at the group or community level					−0.18	−0.22	−0.13	<0.001
ln Health guidance at the individual or group level * ln Health education at the group or community level					−0.07	−0.29	0.15	0.55
sigma_u	0.38				0.35			
sigma_v	1.43E−05				1.27E−08			
Log-likelihood	200.8				264.94			
Likelihood-ratio test value	1.64E+07			<0.001^c^	6.09E+09			<0.001^c^

^a^*The output is the natural log-transformed linearly transformed disability-adjusted life years for unintentional injury per 100,000 population aged under 5 years; higher output values indicate better production of health. The inefficiency terms are assumed to be half-normally distributed*.

^b^*All input variables were centralized after the natural log transformation to avoid multicollinearity*.

^c^*Wald chi-square*.

The translog model yielded similar results to those from the Cobb–Douglas model. Pearson's correlation coefficient for technical efficiency estimated from the Cobb–Douglas and the translog models was 0.85 (*P* < 0.0001).

**Figure 1:**
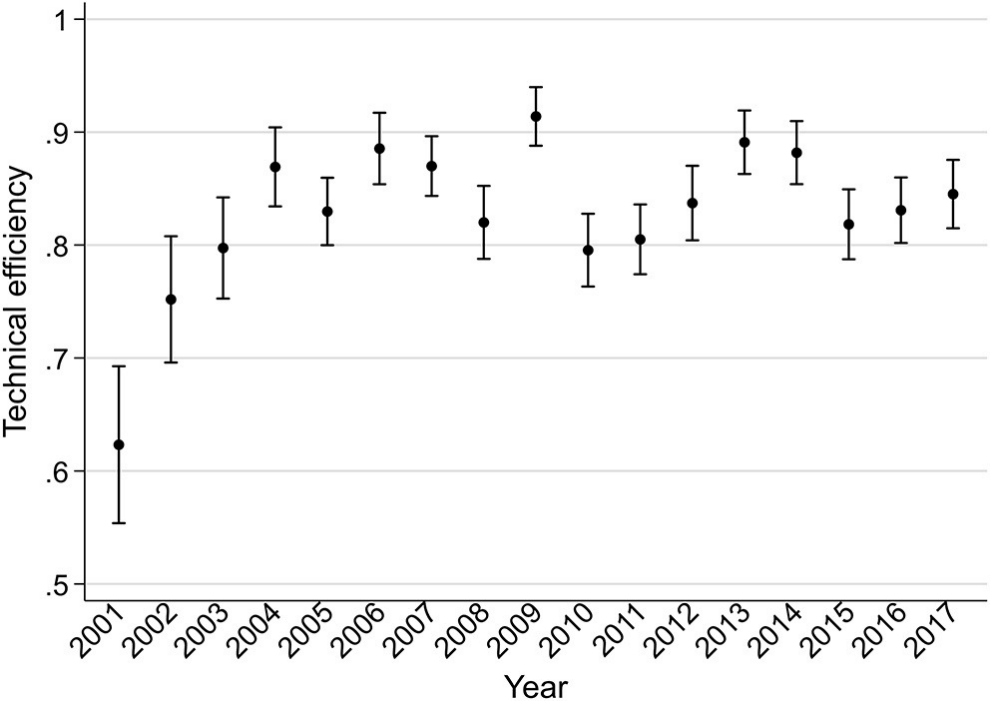
Technical efficiency of 47 prefectures from 2001 to 2017 based on the translog production function. The means of technical efficiency are indicated by point symbols and 95% confidence intervals are indicated by capped bars.

The mean technical efficiency in the 47 prefectures increased from 0.59 in 2001 to 0.85 in 2017 in the Cobb–Douglas model and from 0.62 in 2001 to 0.85 in 2017 in the translog model, as shown in Figure 1. Both models revealed that the efficiency gaps among prefectures narrowed dramatically from 2001 to 2007. However, the narrowing trend stagnated in 2007, and the gaps remained almost constant from that point until 2017. Specifically, in the translog model, the standard deviation of technical efficiency in 2001, 2007, and 2017 was 0.24, 0.09, and 0.10, respectively. The range of the technical efficiency in 2001, 2007, and 2017 was 0.01–1.00, 0.62–1.00, and 0.65–1.00, respectively.

**Figure 2:**
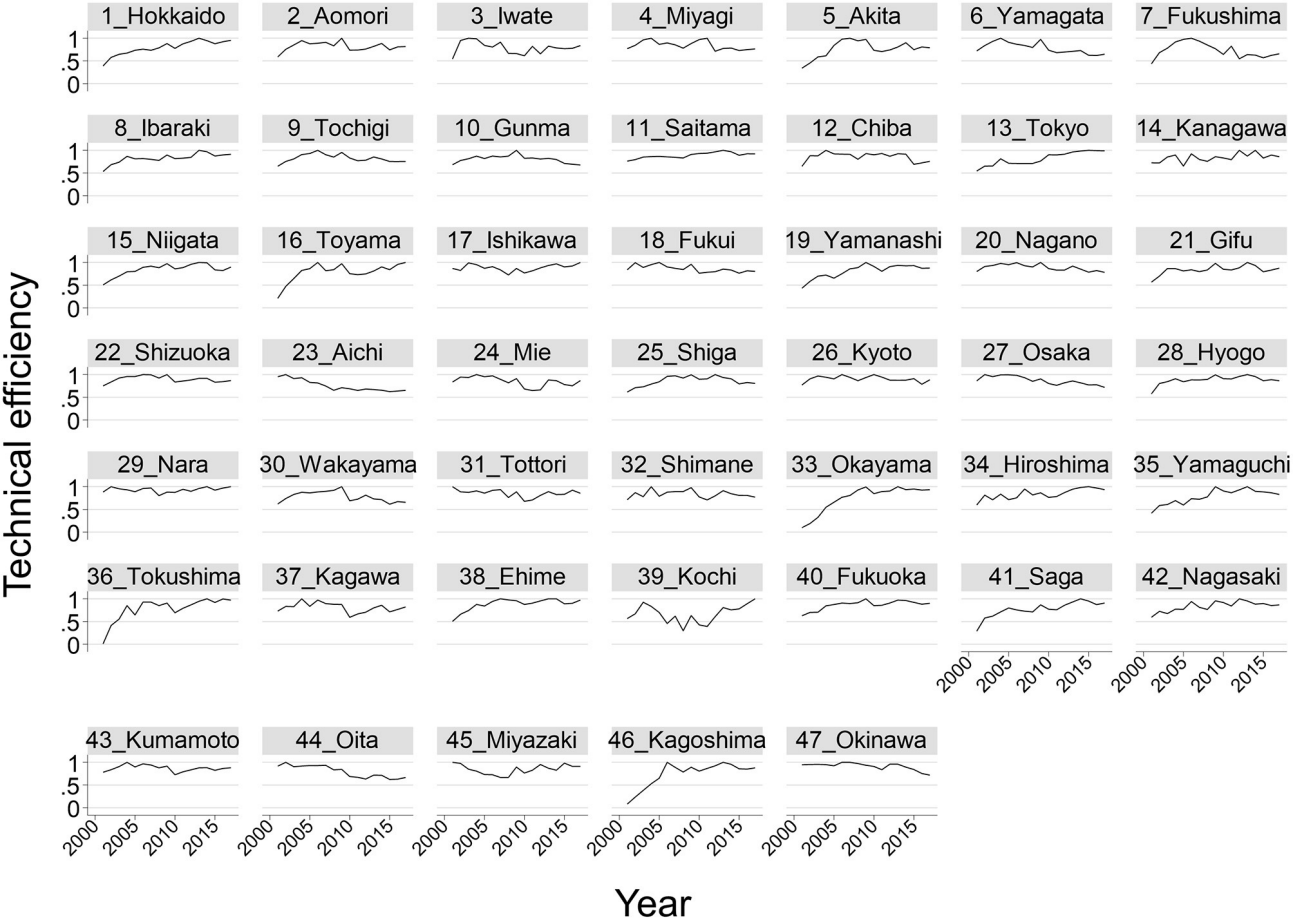
Technical efficiency by prefecture from 2001 to 2017 based on the translog production function. The number listed before each prefecture's name is the prefecture's ID.

The time trend of technical efficiency by prefecture using the translog model is shown in Figure 2. Prefectures with low efficiency (i.e., <0.3) in 2001, such as Toyama, Okayama, Tokushima, Saga, and Kagoshima, reached a high level (i.e., >0.85) in 2017. Conversely, some prefectures, including Yamagata, Fukushima, Aichi, Oita, and Okinawa, showed decreases, reaching the lowest level in 2017. Other prefectures had constant technical efficiency or moderate change in technical efficiency over time.

### Robustness Checks of the Estimation of Technical Efficiency

The estimations were robust to different distributions of the inefficiency terms in the translog model; the Cobb–Douglas models with these distributions did not converge (data not shown; available on request). The translog model assuming interactions between the inputs and constraining factors [Equation (4)] yielded findings that were similar to the main results [Equation (3)] (data not shown; available on request). The results of the estimations excluding areas damaged by the Great East Japan Earthquake were also similar to the main results in the Cobb–Douglas and translog models (data not shown; available on request).

### Contributions of Inputs, Constraints, and Technical Efficiency to National Trends and Regional Disparities in Unintentional Childhood Injury

**Figure 3:**
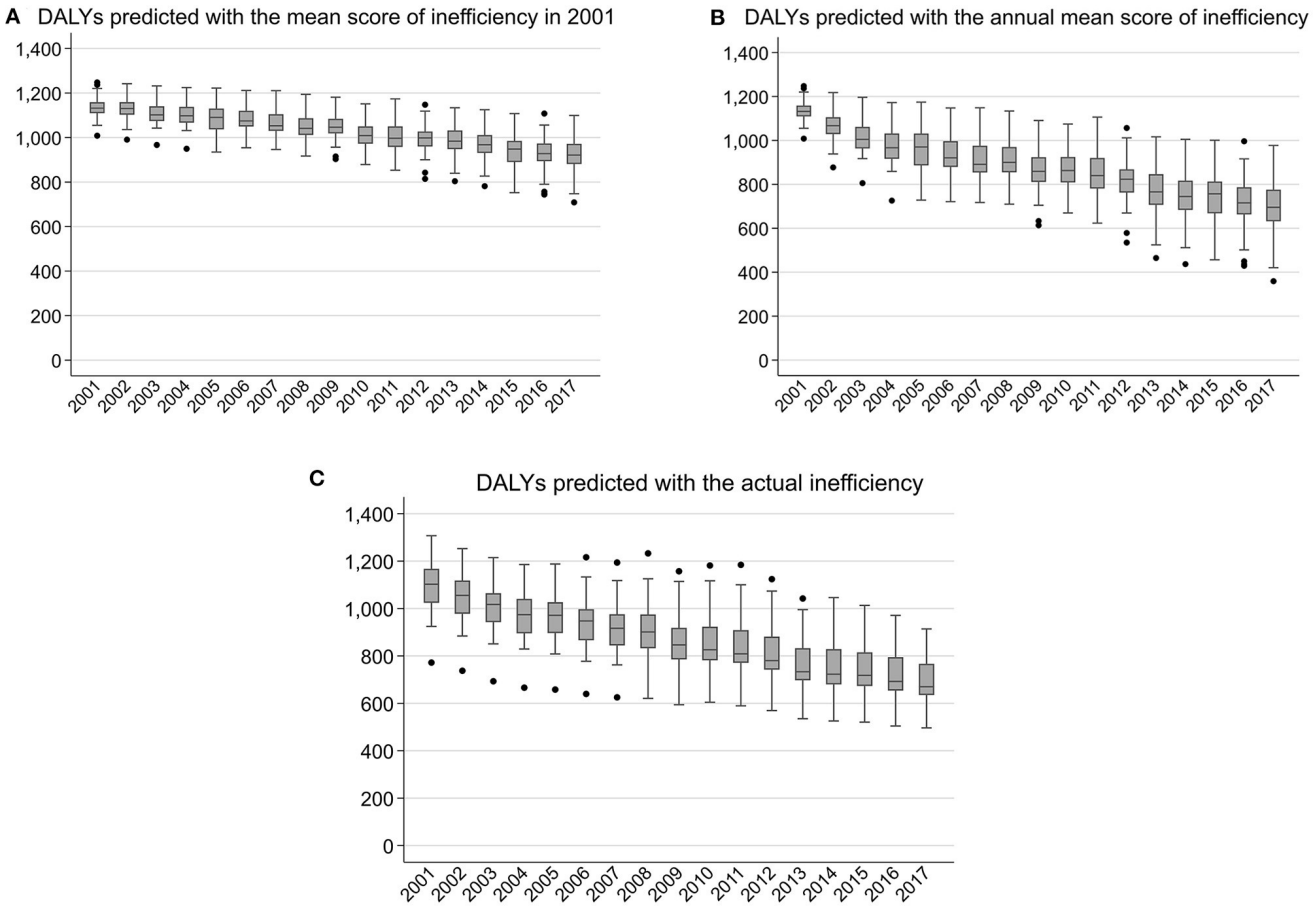
Predicted DALYs from 2001 to 2017 based on the translog production function. DALYs, disability-adjusted life years. DALYs per 100,000 population aged under 5 years.

In the translog model, from 2001 to 2017, the mean DALYs predicted with the mean score of inefficiency in 2001 decreased from 1135.3 to 918.6, and the CV increased from 0.04 to 0.09 (Figure 3A), indicating that national improvements in inputs and constraints decreased the mean DALYs but that the disparities in inputs and constraints widened the regional DALYs gap.

Compared with the trend depicted in Figure 3A, the mean DALYs predicted with the annual mean score of inefficiency showed a steeper decrease, falling from 1135.3 in 2001 to 691.3 in 2017, indicating that the improvement of mean efficiency further contributed to the decrease in mean DALYs (Figure 3B). Figure 3B also shows an increase in the CV from 0.04 in 2001 to 0.19 in 2017, indicating that the regional DALYs gap would have been amplified if mean efficiency improved while inputs and constraints remaining at the current level. However, the CV of DALYs predicted with the actual inefficiency estimates increased only from 0.10 in 2001 to 0.13 in 2017, indicating that the trend of a widening DALYs gap was offset by the actual trajectory of efficiency changes (Figures 3B,C). Specifically, prefectures with low output (high DALYs) improved their efficiency, reaching DALYs lower than those as expected with inputs and constraints. In contrast, prefectures with high output (low DALYs) stagnated efficiency improvement, reaching DALYs higher than those as expected with inputs and constraints. Similar results were obtained with the Cobb–Douglas model (data not shown; available on request).

## Discussion

Our results revealed that the technical efficiency of public health programs has improved and that the disparities in efficiency across prefectures narrowed dramatically until 2007 and then remained constant until 2017. National improvements in inputs, constraints, and technical efficiency contributed to an overall reduction in unintentional childhood injury in Japan. Regional disparities in inputs and constraints widened the population health disparities, but different efficiency improvement trajectories counterbalanced this effect, offsetting the trend of widening health disparities. This study contributes to existing efficiency research by presenting a novel application of efficiency measurement to the assessment of regional health disparity in the context of public health.

### Interpretation of the Estimation of Technical Efficiency

According to a systematic review on the technical efficiency of the overall health systems of OECD countries, Japan has consistently shown the highest efficiency, with an average value of one (42). In this study, however, local disparities were observed in the technical efficiency of public health programs aiming to prevent unintentional childhood injury. Since the 1994 amendment to the Community Health Act, decentralization, or delegating responsibilities to lower-level governments, has strengthened in Japan (17–19). Although decentralization is expected to increase efficiency by using local initiatives for service delivery and procurement budgeting (71), our results suggest that not all local governments make full use of their ability to operate public health programs.

Nevertheless, technical efficiency has improved nationally, and the disparities among prefectures narrowed dramatically from 2001 to 2007. In this study, the mean technical efficiency in the 47 prefectures increased from 0.62 in 2001 to 0.85 in 2017 in the translog model. Similar technical efficiency scores have been reported in studies on local public health sectors in the United States and China (i.e., scores of 0.4–0.7) (50, 51). Although simple comparisons are difficult because of differences in the targeted outputs and the mode of production function, we estimated the average impact of improved technical efficiency on DALYs to be 227.3 DALYs per 100,000 population aged under 5 years from 2001 to 2017.

The national health promotion plan “Healthy Parents and Children 21,” which was initiated in 2001 in Japan (72), is supposed to be one of several nationwide factors reducing efficiency disparities. Because prefectures and municipalities plan and evaluate their public health programs with reference to the national plan, the use of standardized benchmarking for evaluation may reduce the gap in technical efficiency among local governments. Moreover, similar time trends in technical efficiency were observed in most injury categories (data not shown; available on request). The national road safety policies introduced in the first decade of the twenty-first century, such as strengthening punishment for driving under the influence of alcohol and compulsory child restraint seat use (73), could also have reduced the gap in efficiency by enhancing social norms on safety. Nonetheless, the reasons for some prefectures with the lowest levels of efficiency in 2001 seeing drastic improvements in efficiency remain unknown. There is a need for further studies on the program management, political, and organizational factors influencing efficiency.

However, the narrowing trend in the efficiency gaps stagnated in 2007 and subsequently remained constant until 2017. Some prefectures even showed decreased efficiency. Although the exact reasons for these findings are unknown, local governments' prioritization of preventing unintentional injury may have been lowered because of the conflicting demands of other public health programs (e.g., on developmental disability and child abuse prevention) that were newly emphasized because of increased societal awareness (21, 74).

For the production function of unintentional childhood injury, the coefficients of inputs were positive except for the coverage rate of health checkups, which had a negative effect. Although the frequency of health checkups is predetermined by local governments on the basis of policy rather than annual changes in health needs, areas at high risk for childhood injury may promote health staff members' efforts to follow up with children with no visits, which may have led to the negative coefficient of health checkups.

### Policy Implications

Our findings suggest several policy implications. First, public health program efficiency is an important—but ignored—policy target for the SDGs, including closing the regional health gap. To achieve the SDGs, developing strategies based on separate assessments of inputs, constraints, and efficiency is required to identify points of intervention for individual local governments. For example, prefectures with good health outcomes because of high input and an advantageous situation in terms of constraints but low efficiency need to make efforts to improve their efficiency. In contrast, prefectures with poor health outcomes because of low input and a disadvantageous situation in terms of constraints but high efficiency need to increase their input or address their constraints. This study suggests possibilities for creating an assessment system to provide this information using practical regional health sector reports. Additionally, to advocate investments in the SDGs in LMICs, efficiency estimations can be used to produce projections of financial resource needs that are more realistic than the current estimates, which assume efficient practices or simple scenarios (i.e., less efficient or linearly increasing efficiency) (6). Second, with the limited human and financial resources available, policy makers and practitioners should find ways to establish efficient locally operating public health systems. Our estimations suggest that most prefectures in Japan have possibilities for reducing unintentional childhood injury by improving efficiency with the current input level—that is, without increasing expenditures. Further studies are needed to support practitioners to flexibly meet regional demands for the efficient operation of local public health systems.

### The Study's Strengths and Limitations

This study's main strength lies in its estimation of the technical efficiency of preventive public health programs using the production function of health. Our empirical model can be applied to other population health topics where preventive services may have a larger influence than curative medical care, such as dental diseases and frailty among older adults.

The study also had several limitations. First, the calculation of DALYs in this public data source relied on the imputation of missing data on mortality and disease prevalence using the Bayesian meta-regression tool DisMod-MR (64, 75). Our estimation confirmed the high accuracy of the prediction of DALYs because the *R*-squared of the ordinary least squares model regressing original DALYs on DALYs predicted with the actual inefficiency estimates was 1.0. However, the lack of randomness may be attributed to the use of imputation in the DALYs estimation, which may be one of the reasons why the standard deviation of the random error in the TFEM (i.e., sigma_v) was quite small in this study. Although prefecture-level data on mortality from unintentional injuries are available for Japan, we adopted DALYs as the health output because the prevention programs intend to reduce not only death but also injury-related disabilities. Additionally, DALYs have advantages over mortality in that DALYs take into account random errors and deal with the garbage code of deaths. We confirmed that the estimates of efficiency using under-five mortality rate of unintentional injury (Supplementary Table 1 and Supplementary Figure 1) are moderately correlated with the estimations using DAL*Y*s; Pearson's correlation coefficient for technical efficiency was 0.30 (*P* < 0.001). The injury incidence rate could be used as the direct output, but these data were not available. Whether re-estimation with such health outcomes would change the results should be investigated in future studies.

Second, there are other potential inputs in the production function of unintentional childhood injury that were not investigated because of a lack of data availability—namely, programs only targeting families at high risk of adverse child outcomes, prefecture- or municipality-specific parenting support services, and programs with unclear target populations (i.e., optional home visits for children aged over 1 year and health checkups for children aged 4–6 years). The influences of these potential inputs on DALYs, which were included in the fixed effect or inefficiency terms in this study, may be minor because the program scale and target populations are small. Estimations including these programs are needed in future studies. Additionally, public health programs and child protective services are linked, but this study focused solely on the former because the decision-making units for the two types of programs differ (20, 21). Although adjustments for the quality of health care are required in the production function of health care (34, 76), the quality of health care should be included in technical efficiency in the production function of health, given that the output is health status, which relates to care quality. Even though unintentional childhood injury can be attributed primarily to the caregivers' behaviors and the environment, and their impacts may differ by injury categories, it is not feasible to distinguish the contributions of prefectural public health interventions from those of other sources, which may contaminate our estimation of technical efficiency at prefectural levels.

Third, if persistent inefficiency exists, it is completely absorbed in prefecture-specific heterogeneity as fixed effect terms in the TFEM. We believe that the time-variant assumption regarding efficiency in the TFEM (40, 61) is reasonable because the 17-year study period was long enough to observe a change in technical efficiency.

Finally, differences in technical efficiency across municipalities within a single prefecture remain unknown. Although our selection of prefecture as the unit of analysis seems reasonable because it is prefectures that administer public health programs, future studies should investigate efficiency at the municipality level because the programs are operated by municipality-level centers (17–19).

## Conclusions

This study demonstrates that regional disparities exist in the technical efficiency of legally established public health programs aiming to prevent unintentional childhood injury in Japan. The assessment of inputs, constraints, output, and technical efficiency of public health programs could provide policy leverage relevant to region-specific conditions and performance, contributing to the achievement of health promotion and equity.

## Data Availability Statement

Publicly available datasets were analyzed in this study. This data can be found here: “Global Burden of Disease Study 2017” at http://ghdx.healthdata.org/, “Vital statistics 2001–2017” at https://www.mhlw.go.jp/english/database/db-hw/vs01.html, “Population Census 2000, 2005, 2010, 2015” at https://www.stat.go.jp/english/data/kokusei/index.html, “Population estimates 2001–2017” at https://www.stat.go.jp/english/data/jinsui/index.html, “Report on regional public health services and health promotion services 2001–2017” at https://www.mhlw.go.jp/english/database/db-hss/rrphshps.html, “Labour Force Survey 2001–2017” at https://www.stat.go.jp/english/data/roudou/index.html, and “Survey of Medical Institutions 1999–2017” at https://www.mhlw.go.jp/english/database/db-hss/smi.html.

## Author Contributions

AH was responsible for the conception and design of the study, acquisition of the data, analysis and interpretation of the data, drafting the manuscript, and obtaining funding. HK contributed to the analysis and interpretation of the data, provided technical support, and participated in the critical revision of the manuscript. HH was responsible for the conception and design of the study, analysis and interpretation of the data, critical revision of the manuscript, obtaining funding, and providing supervision. All authors approved the final version of the manuscript.

## Funding

This work was supported by the Institute for Health Economics and Policy for fiscal year 2019 (Wakate-kenkyusya-ikusei-kenkyu-josei), and by JSPS KAKENHI Grant Numbers JP21J10730 and JP18H04070. The funding sources had no involvement in the study design, data collection, analysis, interpretation, or preparation of the manuscript.

## Conflict of Interest

The authors declare that the research was conducted in the absence of any commercial or financial relationships that could be construed as a potential conflict of interest.

## Publisher's Note

All claims expressed in this article are solely those of the authors and do not necessarily represent those of their affiliated organizations, or those of the publisher, the editors and the reviewers. Any product that may be evaluated in this article, or claim that may be made by its manufacturer, is not guaranteed or endorsed by the publisher.
