# Supplementary material for: Contribution of the Technical Efficiency of Public Health Programs to National Trends and Regional Disparities in Unintentional Childhood Injury in Japan
Source: Front Public Health. 2022 Jul 12;10:913875. doi: 10.3389/fpubh.2022.913875 (PMC9315066; doi:10.3389/fpubh.2022.913875)
Supplement: Supplementary file 1 [file Data_Sheet_1.pdf]

Supplementary Material

Supplementary Table 1. Estimated production function with under-five mortality rate of unintentional injury using a true fixed effects model (*N* = 47 for 17 years)[a]

	Cobb–Douglas production function				Translog production function[b]			
	Coefficient	95% confidence intervals lower limit	95% confidence intervals upper limit	*P* > |Z|	Coefficient	95% confidence intervals lower limit	95% confidence intervals upper limit	*P* > |Z|
Coverage rates of public health programs								
ln Health checkups for children aged under 1 year	-0.04	-0.52	0.45	0.88				
ln Home visits for children aged under 1 year	0.11	-0.01	0.23	0.07				
ln Health guidance at the individual or group level	0.16	-0.13	0.45	0.27				
ln Health education at the group or community level	0.03	-0.08	0.14	0.60				
ln Proportion of the population with tertiary education	0.11	-0.58	0.80	0.76				
ln Unemployment rate	-0.04	-0.22	0.14	0.69				
ln Density of emergency medical facilities	0.0003	-0.11	0.11	0.99				
ln Population density	-0.24	-1.86	1.37	0.77				
Great East Japan Earthquake dummy	-0.02	-0.57	0.53	0.95				
ln Health checkups for children aged under 1 year (squared)								
ln Home visits for children aged under 1 year (squared)								
ln Health guidance at the individual or group level (squared)								
ln Health education at the group or community level (squared)								
ln Health checkups for children aged under 1 year * ln Home visits for children aged under 1 year								
ln Health checkups for children aged under 1 year * ln Health guidance at the individual or group level								

ln Health checkups for children aged under 1 year * ln Health education at the group or community level			
ln Home visits for children aged under 1 year * ln Health guidance at the individual or group level			
ln Home visits for children aged under 1 year * ln Health education at the group or community level			
ln Health guidance at the individual or group level * ln Health education at the group or community level			
sigma_u	0.47		
sigma_v	2.90E-10		
Log-likelihood	21.2		
Likelihood-ratio test value	22.66	<0.01[c]	

[a]The output is the natural log-transformed linearly transformed under-five mortality rate of unintentional injury (the number of deaths of unintentional injury per 100 000 population aged under 5 years); higher output values indicate better production of health. Other unintentional injuries, which include injuries by exposure to forces of nature, were excluded from the analysis. The inefficiency terms are assumed to be half-normally distributed.
[b]Translog production function did not converge.
[c]Wald chi-square

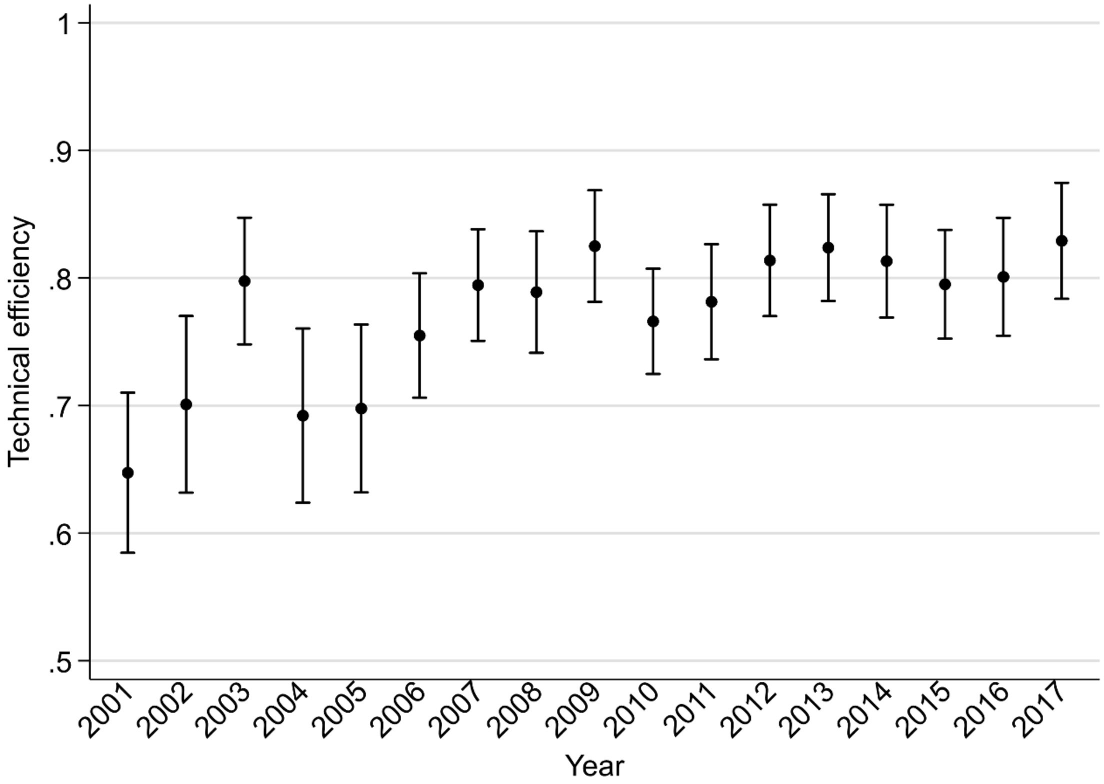

Supplementary Figure 1. Technical efficiency of 47 prefectures from 2001 to 2017 based on the Cobb-Douglas production function using under-five mortality rate of unintentional injury as output

The means of technical efficiency are indicated by point symbols and 95% confidence intervals are indicated by capped bars.
